# Frequency of discussing and documenting advance care planning in primary care: secondary analysis of a multicenter cross-sectional observational study

**DOI:** 10.1186/s12904-020-00543-y

**Published:** 2020-03-17

**Authors:** Jun Hamano, Ai Oishi, Tatsuya Morita, Yoshiyuki Kizawa

**Affiliations:** 1 Division of Clinical Medicine, Faculty of Medicine, University of Tsukuba, 1-1-1 Tennoudai, Tsukuba, Ibaraki, 305-8575 Japan; 2 Primary Palliative Care Research Group, Usher Institute of Population Health Sciences and Informatics, University of Edinburgh, Medical School (Doorway 1), Teviot Place, Edinburgh, UK; 3 Department of Palliative and Supportive Care, Palliative Care Team, and Seirei Hospice, Seirei Mikatahara General Hospital, 3453 Mikatahara-cho, Kita-ku, Hamamatsu, Shizuoka Japan; 4 Department of Palliative Medicine, Kobe University Graduate School of Medicine, Kobe, Hyogo Japan

**Keywords:** Advance care planning, Aged patients, End of life care, Family physician, Primary care outpatients, Supportive and palliative care indicators tool

## Abstract

**Background:**

To improve the quality of advance care planning (ACP) in primary care, it is important to understand the frequency of and topics involved in the ACP discussion between patients and their family physicians (FPs).

**Methods:**

A secondary analysis of a previous multicenter cross-sectional observational study was performed. The primary outcome of this analysis was the frequency of and topics involved in the ACP discussion between outpatients and FPs. In March 2017, 22 family physicians at 17 clinics scheduled a day to assess outpatients and enrolled patients older than 65 years who were recognized by FPs as having regular visits. We defined three ACP discussion topics: 1) future decline in activities of daily living (ADL), 2) future inability to eat, and 3) surrogate decision makers. FPs assessed whether they had ever discussed any ACP topics with each patient and their family members, and if they had documented the results of these discussions in medical records before patients were enrolled in the present study. We defined patients as being at risk of deteriorating and dying if they had at least 2 positive general indicators or at least 1 positive disease-specific indicator in the Japanese version of the Supportive and Palliative Care Indicators Tool.

**Results:**

In total, 382 patients with a mean age of 77.4 ± 7.9 years were enrolled, and 63.1% were female. Seventy-nine patients (20.7%) had discussed at least one ACP topic with their FPs. However, only 23 patients (6.0%) had discussed an ACP topic with family members and their FPs, with the results being documented in their medical records. The topic of future ADL decline was discussed and documented more often than the other two topics. Patients at risk of deteriorating and dying discussed ACP topics significantly more often than those not at risk of deteriorating and dying (39.4% vs. 16.8%, *p* < 0.001).

**Conclusion:**

FPs may discuss ACP with some of their patients, but may not often document the results of this discussion in medical records. FPs need to be encouraged to discuss ACP with patients and family members and describe the decisions reached in medical records.

## Introduction

Advance care planning (ACP) is an important process for sharing care preferences, discussing the goals of care, and making care plans through discussions between patients and health care providers [1, 2]. A recent study revealed that appropriate ACP was beneficial for patients, their families, and the health system because it increases autonomy, dignity, peace, and intimacy at the time of death, lessens grieving and decreases the risk of mental health issues for family members, and reduces health care costs and the utilization of resources [3].

Previous studies indicated that early recognition of the risk of a particular patient deteriorating and dying is difficult, which suggests that the opportunity for ACP discussions needs to be provided at the right time from the perspective of illness trajectories [4, 5]. Since illness trajectories vary widely, the development of ACP that considers an individual patient’s trajectory is important [5].

In the opinion of patients, families, and health care professionals, the family physician (FP) is the key professional for the discussion of ACP [4, 6–9]. Previous studies investigated the attitude of FPs toward ACP and the facilitators of or barriers to discussing ACP with FPs [10]; limited information is currently available on frequency of and topics involved in the ACP discussion among patients, their family members, and FPs [11–17].

According to a previous mortality follow-back study, 34% of 1072 patients with non-sudden death had discussed ACP with their FPs [16]. That study focused on terminally ill patients, and revealed that the most frequent topic of discussion was not adopting potential life-prolonging treatments. Another cross-sectional study revealed that ACP was discussed between 16.3% of outpatients and their FPs; however, the topics under discussion were not investigated [17]. In addition, a recent study based on hypothetical vignette scenarios showed that FPs were more likely to discuss ACP when patients had severe clinical manifestations [10]. A better understanding of the frequency of and topics involved in the ACP discussion as well as the characteristics of patients who discuss ACP with their FPs may contribute to improving ACP quality in the primary care setting.

Therefore, we herein performed a secondary analysis of our previous multicenter observational study to investigate the frequency of and topics involved in the ACP discussion among outpatients, their family members, and FPs involved in primary care. In addition, the frequency of the ACP discussion was compared between patients who were/were not at risk of deteriorating and dying, and the relationships between FP background factors and the discussion of ACP were examined.

## Methods

The present study involved a secondary analysis of our previous multicenter cross-sectional observational study in Japan. Our previous study focused on the prevalence and characteristics of primary care outpatients at risk of deteriorating and dying [18]. Participating facilities and FPs were collected by purposive sampling. Each participating clinic provided ambulatory care for community residents and had at least one FP. In March 2017, 17 clinics (22 FPs) scheduled a day to assess their outpatients in advance. Patients who were older than 65 years and who were recognized by FPs as having regular visits were enrolled. The present study was approved by the Institutional Review Board of the University of Tsukuba (No. 1089).

### Advance care planning

ACP was defined according to a previous Delphi study that indicated ACP as a process that supports adults of any age or health status to understand and share their personal values, life goals, and preferences regarding future medical care [2]. We also defined three ACP discussion topics, and developed ad hoc questionnaires based on a literature review and discussion among the authors [1, 2, 11, 12, 14, 16, 17, 19, 20]. The three ACP discussion topics were as follows: 1) future decline in the activities of daily living (ADL), 2) future inability to eat, and 3) surrogate decision makers. We defined the frequency of the ACP discussion as discussing any of these topics at least once among the patient and FP before enrollment in the study. Even when patients and FPs talked about multiple topics more than once, it was considered to be one ACP discussion. In addition, we defined an optimized ACP discussion as a discussion held among the patient, family members, and FP together with documentation of the results in medical records. This definition of an optimized ACP discussion was based on the international consensus definition of ACP, namely, ACP involves a patient discussing the above-mentioned goals and preferences with family members and health care providers, followed by recording and reviewing the patient’s preferences if appropriate [1].

### Patients at risk of deteriorating and dying

We identified patients who were at risk of deteriorating and dying using the Japanese version of the Supportive and Palliative Care Indicators Tool (SPICT-JP), as previously reported [18]. The original Supportive and Palliative Care Indicators Tool (SPICT™) was developed to identify patients whose health condition is deteriorating. Details of the development of SPICT™ and SPICT-JP have been described elsewhere [18, 21–25].

### Data collection

FPs were asked whether they had ever discussed any of the ACP topics (a patient’s future ADL decline, future inability to eat, and surrogate decision makers) with each patient and their family members before the patient was enrolled in the present study based on the FP’s memory, and to confirm whether the discussion had been documented in medical records. FPs also assessed patients according to the Palliative Performance Scale (PPS) [26], and 6 general clinical indicators and 25 disease-specific indicators in the SPICT-JP (Additional file 1). Furthermore, FPs recorded the demographic and clinical characteristics of patients. We also investigated the background factors of FPs, including sex, duration of clinical practice after obtaining a medical license, experience with a palliative care unit, and participation in the nationwide palliative care education program.

### Statistical analysis

Descriptive statistics were calculated for each ACP discussion topic. According to previous studies, we defined patients as being at risk of deteriorating and dying if they had at least 2 positive general indicators or at least 1 positive disease-specific indicator in the SPICT-JP [25]. The relationship between the discussion of ACP and a patient’s risk of deteriorating and dying was examined. We also investigated the relationship between the discussion of ACP and patients with PPS ≤ 70. The characteristics of participants were reported as proportions for categorical variables and were analyzed by Pearson’s χ2 test or Fisher’s exact test, while continuous variables were analyzed by the Student’s *t*-test. To examine the relationship between FP background factors and the discussion of ACP topics, we performed univariate and multivariate logistic regression analyses to calculate the odds ratio (OR) and 95% confidence interval (95% CI). FPs were categorized into 2 groups based on the duration of clinical practice (> 15 years or ≤ 15 years). Background factors of FPs that showed significance in univariate analyses were employed as independent variables in multivariate analyses. SPSS-J software (version 24.0; IBM, Tokyo, Japan) was used to conduct all analyses, and *p* < 0.05 was considered to be significant.

## Results

In total, 382 patients from 17 clinics (22 FPs) were included. Their mean age was 77.4 ± 7.9 years, and 63.1% were female. Most patients had PPS ≥ 80 (79.1%), and did not use care services (81.4%). The main underlying diseases were hypertension (31.9%), dementia/frailty (15.2%), and cardiovascular disease, excluding hypertension (9.2%) (Table 1).

**Table 1:** Demographic and Clinical Characteristics of Patients (*n* = 382)

	n	%
Age (mean ± standard deviation)	77.4 ± 7.9	
Sex		
Male	141	36.9
Female	241	63.1
Living situation		
Living with family	298	78.0
Living alone	59	15.4
Care facility	8	2.1
Main underlying disease		
Hypertension	122	31.9
Dementia/frailty	58	15.2
Cardiovascular disease (excluding hypertension)	38	9.9
Diabetes	30	7.9
Hyperlipidemia	19	5.0
Neurological disease	18	4.7
Cancer	14	3.7
Respiratory disease	13	3.4
Musculoskeletal disease	8	2.1
Mental disorder	6	1.6
Gastroesophageal reflux disease	6	1.6
Kidney disease	5	1.3
Liver disease	3	0.8
Others	42	11.0
Palliative performance scale		
100	202	52.9
90	51	13.4
80	49	12.8
70	20	5.2
60	33	8.6
50	22	5.8
40	5	1.3
Current use of care services		
No care service	311	81.4
One or more care services	71	18.6
Types of care services used (Multiple answers)^a^		
Home visit nursing	11	2.9
Nursing care services	16	4.2
Home visit pharmacist	1	0.3
Day care service	54	14.1
Specialized palliative care service	2	0.5

^a^The type of care service used involved multiple choice questions, and most patients did not use the care services

Among the 22 participating physicians, 12 had trained at a palliative care unit (54.5%) and 17 had participated in the nationwide palliative care education program (77.3%) [27, 28] (Additional file 2).

### Frequency of the ACP discussion and topics

While 20.7% of patients had discussed at least one of the ACP topics with their FPs, only 6.0% had participated in an optimized ACP discussion (Table 2).

**Table 2:** Comparison of the frequency and topics of ACP about patients being at risk of deteriorating and dying, and about patients with Palliative Performance Scale = 70 or less

	All patients (*n* = 382)		Patients at risk of deteriorating and dying^c^ (*n* = 66)		Patients not at risk of deteriorating and dying^d^ (*n* = 316)		p	Palliative performance scale = 70 or less (*n* = 80)		Palliative performance scale = 80 or above (*n* = 302)		p
	n	%	n	%	n	%		n	%	n	%	
About future ADL decline												
Discussed with the patient	69	18.1	19	28.8	50	15.8	0.013	17	21.3	52	17.2	0.416
Discussed with the family	28	7.3	16	24.2	12	3.8	< 0.001^a^	19	23.8	9	3.0	< 0.001
Documented in medical records	46	12.0	14	21.2	32	10.1	0.012	12	15.0	34	11.3	0.439
Discussed with the patient and documented in medical records	30	7.9	6	9.1	24	7.6	0.681	2	2.5	28	9.3	0.059
Discussed with the family and documented in medical records	1	0.3	1	1.5	0	0.0	0.173^a^	1	1.3	0	0.0	0.209^a^
Discussed with the patient and family and documented in medical records	15	3.9	7	10.6	8	2.5	0.007^a^	9	11.3	6	2.0	0.001^a^
About future inability to eat												
Discussed with the patient	38	9.9	10	15.2	28	8.9	0.120	6	7.5	32	10.6	0.086
Discussed with the family	14	3.7	7	10.6	7	2.2	0.004^a^	7	8.8	7	2.3	0.013^a^
Documented in medical records	24	6.3	5	7.6	19	6.0	0.583^a^	2	2.5	22	7.3	0.130
Discussed with the patient and documented in medical records	17	4.5	3	4.5	14	4.4	1.000^a^	0	0.0	17	5.6	0.29^a^
Discussed with the family and documented in medical records	0	0.0	0	0.0	0	0.0	n.a	0	0.0	0	0.0	n.a
Discussed with the patient and family and documented in medical records	7	1.8	2	3.0	5	1.6	0.348^a^	2	2.5	5	1.7	0.640^a^
About surrogate decision makers												
Discussed with the patient	32	8.4	11	16.7	21	6.6	0.008	12	15.0	20	6.6	0.023
Discussed with the family	21	5.5	12	18.2	9	2.8	< 0.001^a^	15	18.8	6	2.0	< 0.001^a^
Documented in medical records	24	6.3	7	10.6	17	5.4	0.157^a^	9	11.3	15	5.0	0.065
Discussed with the patient and documented in medical records	10	2.6	0	0.0	10	3.2	0.222^a^	0	0.0	10	3.3	0.130^a^
Discussed with the family and documented in medical records	0	0.0	0	0.0	0	0.0	n.a	0	0.0	0	0.0	n.a
Discussed with the patient and family and documented in medical records	14	3.7	7	10.6	7	2.2	0.004^a^	9	11.3	5	1.7	< 0.001^a^
About any one ACP topic^b^												
Discussed with the patient	79	20.7	26	39.4	53	16.8	< 0.001	24	30.0	55	18.2	0.029
Discussed with the family	36	9.4	22	33.3	14	4.4	< 0.001	26	32.5	10	3.3	< 0.001
Documented in medical records	56	14.7	20	30.3	36	11.4	< 0.001	19	23.8	37	12.3	0.013
Discussed with the patient and documented in medical records	34	8.9	6	9.1	28	8.9	1.000	2	2.5	32	10.6	0.025
Discussed with the family and documented in medical records	1	0.3	1	1.5	0	0.0	< 0.173^a^	1	1.3	0	0.0	0.209^a^
Discussed with the patient and family and documented in medical records	23	6.0	13	19.7	10	3.2	< 0.001	16	20.0	7	2.3	< 0.001^a^

^a^Fisher’s exact test

^b^ACP topics were; future ADL declines, future inability to eat, and surrogate decision makers

^c^With two or more general indicators or one or more clinical indicators by the SPICT-JP

^d^Without two or more general indicators or one or more clinical indicators by the SPICT-JP

Patients with liver disease had never discussed any of the ACP topics. On the other hand, one third of patients with cancer and neurological diseases, and one fifth of those with dementia/frailty had discussed at least one topic (Additional file 3). Each of the three ACP topics was discussed more frequently with patients (8.4–18.1%) than with their family members (3.7–7.3%) (Table 2). The topic of future ADL decline was discussed and documented more often than the other two topics. In 56 out of 79 patients (70.9%) who had discussed an ACP topic, the discussion was documented in their medical records.

### Distribution of indicators for deteriorating and dying

The distribution of general and specific indicators for deteriorating health based on the SPICT-JP is shown in Table 3. The most frequent general indicator was “The patient or family asked for palliative care, treatment withdrawal/limitation, or a focus on quality of life” (25.4%). Based on our definition, 66 patients (17.3%) were identified as being at risk of deteriorating and dying.

**Table 3:** Prevalence of patients at risk of deteriorating and dying

	n	%
General clinical risk of deteriorating health (*n* = 382)		
Two or more unplanned hospital admissions in the past 6 months	1	0.3
Performance status is poor or deteriorating, with limited reversibility	24	6.3
Dependent on others for care due to physical and/or mental health issues	26	6.8
Significant weight loss over the past 3–6 months and/or a low body mass index	18	4.7
Persistent symptoms despite optimal treatment of the underlying condition(s)	16	4.2
The patient or family asked for palliative care, treatment withdrawal/limitation, or a focus on quality of life	97	25.4
Disease-specific risk for the deterioration of the conditions		
Cancer (*n* = 14)		
Functional ability deteriorating due to progressive cancer	3	21.4
Too frail for cancer treatment or treatment for symptom control	2	14.3
Dementia/frailty (*n* = 56)		
Unable to dress, walk, or eat without help	12	21.4
Eating and drinking less; difficulty swallowing	9	16.1
Urinary and fecal incontinence	13	23.2
No longer able to communicate using verbal language; little social interaction	23	41.1
Fractured femur; multiple falls	10	17.9
Recurrent febrile episodes or infections; aspiration pneumonia	2	3.6
Neurological disease (*n* = 18)		
Progressive deterioration of physical and/or cognitive function despite optimal therapy	7	38.9
Speech problems with increasing difficulty communicating and/or progressive difficulty swallowing	2	11.1
Recurrent aspiration pneumonia; breathless or respiratory failure	1	5.6
Cardiovascular disease (*n* = 38)		
NYHA Class III/IV heart failure or extensive, untreatable coronary artery disease with breathlessness or chest pain at rest or on minimal exertion	4	10.5
Severe, inoperable peripheral vascular disease	0	0.0
Respiratory disease (*n* = 13)		
Severe chronic lung disease with breathlessness at rest or on minimal exertion between exacerbations	2	15.4
Needs long-term oxygen therapy	1	7.7
Has needed ventilation for respiratory failure or ventilation is contraindicated	0	0.0
Kidney disease (*n* = 5)		
Stage 4 or 5 chronic kidney disease (eGFR < 30 ml/min) with deteriorating health	3	60.0
Kidney failure complicating other life-limiting conditions or treatments	3	60.0
Stopping dialysis	0	0.0
Liver disease (*n* = 3)		
Advanced cirrhosis with one or more complications in the past year: diuretic-resistant ascites	0	0.0
Advanced cirrhosis with one or more complications in the past year: hepatic encephalopathy	0	0.0
Advanced cirrhosis with one or more complications in the past year: hepatorenal syndrome	0	0.0
Advanced cirrhosis with one or more complications in the past year: bacterial peritonitis	0	0.0
Advanced cirrhosis with one or more complications in the past year: recurrent variceal bleeds	0	0.0
Liver transplantation is contraindicated	2	66.7

### Frequency of the ACP discussion and topics among patients at risk of deteriorating and dying, and patients with PPS ≤ 70

Table 2 shows the frequency of the ACP discussion and discussion topics among patients at risk of deteriorating and dying and patients with PPS ≤ 70. These patients discussed any one of the ACP topics significantly more often than those not at risk of deteriorating and dying (39.4% vs. 16.8%, *p* < 0.001), and also had a significantly higher frequency of an optimized ACP discussion (19.7% vs. 3.2%, p < 0.001).

The topics of future ADL decline and surrogate decision makers were discussed significantly more often with patients at risk of deteriorating and dying than with those who were not at risk (28.8% vs. 15.8%, *p* = 0.013; 16.7% vs. 6.6%, *p* = 0.008); however, no significant differences were observed in future inability to eat (15.2% vs. 8.9%, *p* = 0.12). An optimized ACP discussion on future ADL decline and surrogate decision makers was significantly more frequent among patients at risk of deteriorating and dying than among those not at risk (10.6% vs. 2.5%, *p* = 0.007; 10.6% vs. 2.2%, *p* = 0.004).

Patients with PPS ≤ 70 (*n* = 80) discussed any ACP topic significantly more often than those with PPS ≥ 80 (30.0% vs. 18.2%, *p* = 0.029), and had a significantly higher frequency of the optimized ACP discussion (20.0% vs. 2.3%, *p* < 0.001).

### FP backgrounds and discussion of ACP

According to the univariate analysis, male sex (OR = 8.0, 95%CI 1.9–33.5, *p* = 0.001), clinical practice for ≥15 years (OR = 2.1, 95%CI 1.3–3.4, *p* = 0.005), training in a palliative care unit (OR = 2.1, 95%CI 1.3–3.5, p = 0.005), and participation in the nationwide palliative care education program (OR = 2.1, 95%CI 1.0–4.3, *p* = 0.044) correlated with the discussion of ACP topics. The multivariate analysis confirmed that male sex (OR = 6.6, 95%CI 1.5–29.3, *p* = 0.012), clinical practice for ≥15 years (OR = 1.9, 95%CI 1.1–3.3, *p* = 0.021), and training in a palliative care unit (OR = 2.6, 95%CI 1.4–4.6, *p* = 0.002) correlated with the discussion of ACP topics (Additional file 4).

## Discussion

To the best of our knowledge, this is the first large-scale cross-sectional survey on the frequency of discussing ACP and the topics involved among primary care outpatients, their family members, and FPs. The first important result was that 20.7% of primary care outpatients aged > 65 years had discussed at least one ACP topic with their FPs. However, only 6% of patients had discussed at least one topic with their family members and FPs with documentation of the discussion in their medical records.

Since the frequency of discussing ACP depends on patient background factors and the definition of the discussion, a systematic review revealed that its frequency among frail elderly patients ranged widely between 2 and 29% [13]. When limited to studies with a similar patient background and definition of discussing ACP, the present results were consistent with a multicenter cross-sectional study on primary care outpatients in Japan, in which the frequency of discussing ACP was 16.2% [17]. Therefore, approximately one fifth of primary care outpatients may participate in ACP discussions on future health care or surrogate decision makers with their FPs. It is difficult to judge whether this represents an appropriate discussion of ACP because the quality of end-of-life care was not assessed in the present study.

ACP discussions among patients, family members, and FPs with documentation in medical records, which we defined as an optimized ACP discussion, have not yet been examined. Considering the purpose of discussing ACP, it appears to be important to perform the optimized discussion in order to improve the quality of end-of-life care. We found that only 6.0% of patients had the optimized ACP discussion, suggesting effective ACP discussion processes have not yet been implemented in primary care.

The second important result of the present study was that almost one fifth of patients had discussed future ADL decline with their FPs. Although few studies have investigated the frequency of ACP discussion topics in the primary care setting, this result is similar to a previous finding showing that only 17% of community dwelling persons older than 80 years had discussed their wishes for end-of-life care with a physician or health care provider [20].

On the other hand, 42% of patients with mild dementia discussed illness-related topics, while 34% discussed preferences for medical treatment with their FPs during the last 3 months of life [11]. This difference from the present results may be attributed to the frequency of discussing ACP increasing with the risk of deteriorating and dying because a previous study using hypothetical vignette scenarios revealed that FPs identified patients with severe clinical manifestations as needing the ACP discussion [10].

The third important result was that the discussion of ACP topics was significantly more frequent among patients at risk of deteriorating and dying than among those who were not at risk. This result is consistent with the study based on hypothetical vignette scenarios, which revealed that FPs were more likely to discuss ACP with patients showing severe clinical features [10]. Since the SPICT-JP was not assessed in the present study, our results may reflect the opinions of FPs on the risk of deteriorating and dying for primary care outpatients.

It is important to note that patients with PPS ≤ 70 had a significantly higher proportion of ACP discussions with patient (*p* = 0.029). However, the proportion of discussions with patients documented in medical records about any one of the ACP topics was significantly lower in patients with PPS ≤ 70 (*p* = 0.025), whereas no significant difference was observed among patients at risk of deteriorating and dying or not (*p* = 1.000). This result suggests that FPs conduct an ACP discussion based on factors other than a poor performance status, such as a patient or family member asking for palliative care, treatment withdrawal/limitation, or a focus on quality of life.

The fourth important result was that male FPs with long clinical experience and training at palliative care units were more proactive about discussing ACP; however, the present results were not adjusted for variables such as the perception of ACP and end-of-life care. This result is consistent with the findings of a systematic review, which indicated that accumulated skills facilitate the engagement of FPs in ACP discussions [29]. While the present study suggested that male FPs were more frequently involved in ACP discussions, Fulmer et al. reported that female physicians were more likely to have these discussions [30]. This difference may have arisen because Fulmer’s study included physicians from several specialties working in the hospital setting as well as FPs. Thus, further studies are needed to investigate the background factors of FPs that influence discussions of ACP in the primary care setting.

The present study had several limitations. We targeted a very small proportion of certified FPs in Japan by purposive sampling; therefore, the results obtained may not be representative and their interpretation requires caution. Furthermore, the present results may have been influenced by the Japanese health care system and cultural background; therefore, difficulties are associated with generalizing these results to other countries. In addition, observer recall bias may have influenced the data obtained because difficulties are associated with ascertaining whether undocumented ACP discussions occurred. A gap may exist in the perception of the ACP discussion between FPs and their patients. Another limitation is that there is currently no consensus that the three ACP discussion topics defined in the present study are standard evaluation items of the ACP process and outcomes. Therefore, caution is required when assessing the ACP process and outcomes performed by FPs based on the results of this study.

## Conclusion

FPs may only discuss ACP with a few of their patients; however, this discussion may be more frequent with patients who are at risk of deteriorating and dying. The topic of future ADL decline was discussed and documented more often than other topics. However, FPs may not document the results of most ACP discussions in medical records. Further investigations are needed to establish whether the discussion of ACP between patients and FPs improves the quality of end-of-life care.

## Supplementary information

**Additional file 1.** Supportive and Palliative Care Indicators Tool (SPICT™), April 2015.

**Additional file 2 **Backgrounds of participating physicians (*n* = 22).

**Additional file 3.** Prevalence of the ACP discussion and topics discussed in each disease category.

**Additional file 4.** Relationships between physician background factors and the ACP discussion.

## Data Availability

The datasets used and/or analyzed during the present study are available from the corresponding author on reasonable request.

## Abbreviations

ACP: Advance care planning
FPs: Family physicians
SPICT-JP: The Japanese version of the Supportive and Palliative Care Indicators Tool
SPICT: The Supportive and Palliative Care Indicators Tool
PPS: Palliative Performance Scale
OR: Odds ratio
95% CI: 95% confidence interval
