# Supplementary material for: Frequency of discussing and documenting advance care planning in primary care: secondary analysis of a multicenter cross-sectional observational study
Source: BMC Palliat Care. 2020 Mar 17;19:32. doi: 10.1186/s12904-020-00543-y (PMC7079526; doi:10.1186/s12904-020-00543-y)
Supplement: Supplementary file 1 — Additional file 1. Supportive and Palliative Care Indicators Tool (SPICT™), April 2015. [file 12904_2020_543_MOESM1_ESM.docx]

Additional file 1 Supportive and Palliative Care Indicators Tool (SPICT™), April 2015

| **Look for two or more general indicators of deteriorating health** | | | | |  |  |  |  |
| --- | --- | --- | --- | --- | --- | --- | --- | --- |
| • Performance status is poor or deteriorating (in bed or a chair for ≥50% of the day); reversibility is limited | | | | | | | | |
| • Dependent on others for most care needs due to physical or mental health issues | | | | | | |  |  |
| • Two or more unplanned hospital admissions in the past 6 months | | | | |  |  |  |  |
| • Significant weight loss (5–10%) over the past 3–6 months or a low body mass index | | | | | | |  |  |
| • Persistent, troublesome symptoms despite optimal treatment of the underlying condition(s) | | | | | | |  |  |
| • Patient asks for supportive and palliative care or treatment withdrawal | | | | | |  |  |  |
|  |  |  |  |  |  |  |  |  |
| **Look for any clinical indicators of one or more advanced conditions** | | | | |  |  |  |  |
| **Cancer** |  |  |  |  |  |  |  |  |
| • Functional ability deteriorating due to progressive metastatic cancer | | | | |  |  |  |  |
| • Too frail for oncology treatment or treatment for symptom control | | | | |  |  |  |  |
| **Dementia/frailty** | |  |  |  |  |  |  |  |
| • Unable to dress, walk, or eat without help | | |  |  |  |  |  |  |
| • Eating and drinking less or swallowing difficulties | | | |  |  |  |  |  |
| • Urinary and fecal incontinence | | |  |  |  |  |  |  |
| • No longer able to communicate using verbal language or little social interaction | | | | | |  |  |  |
| • Femur fracture or multiple falls | | |  |  |  |  |  |  |
| • Recurrent febrile episodes or infections, or aspiration pneumonia | | | | |  |  |  |  |
| **Neurological diseases** | |  |  |  |  |  |  |  |
| • Progressive deterioration in physical or cognitive function despite optimal therapy | | | | | | |  |  |
| • Speech issues with increasing difficulty communicating or progressive swallowing difficulties | | | | | | |  |  |
| • Recurrent aspiration pneumonia, breathlessness, or respiratory failure | | | | |  |  |  |  |
| **Heart/vascular disease** | |  |  |  |  |  |  |  |
| • NYHA* Class III/IV heart failure or extensive untreatable coronary artery disease with breathlessness or chest pain at rest or on minimal exertion | | | | | |  |  |  |
| • Severe inoperable peripheral vascular disease | | | |  |  |  |  |  |
| **Respiratory disease** | |  |  |  |  |  |  |  |
| • Severe chronic lung disease with breathlessness at rest or on minimal exertion between exacerbations | | |  |  |  |  |  |  |
| • Needs long-term oxygen therapy | | |  |  |  |  |  |  |
| • Has needed ventilation for respiratory failure or ventilation is contraindicated | | | | | |  |  |  |
| **Kidney disease** | |  |  |  |  |  |  |  |
| • Stage 4 or 5 chronic kidney disease (eGFR#< 30 ml/min) with deteriorating health | | | | | |  |  |  |
| • Kidney failure complicating other life-limiting conditions or treatments | | | | |  |  |  |  |
| • Discontinuation of dialysis | |  |  |  |  |  |  |  |
| **Liver disease** | |  |  |  |  |  |  |  |
| • Advanced cirrhosis with one or more complications in the past year: | | | | |  |  |  |  |
| Diuretic-resistant ascites | |  |  |  |  |  |  |  |
| Hepatic encephalopathy | |  |  |  |  |  |  |  |
| Hepatorenal syndrome | |  |  |  |  |  |  |  |
| Bacterial peritonitis | |  |  |  |  |  |  |  |
| Recurrent variceal bleeding | |  |  |  |  |  |  |  |
| • Liver transplantation is contraindicated | | |  |  |  |  |  |  |
| *NYHA: New York Heart Association | | |  |  |  |  |  |  |
| #eGFR: estimated glomerular filtration rate | | |  |  |  |  |  |  |
