# Supplementary material for: Frequency of discussing and documenting advance care planning in primary care: secondary analysis of a multicenter cross-sectional observational study
Source: BMC Palliat Care. 2020 Mar 17;19:32. doi: 10.1186/s12904-020-00543-y (PMC7079526; doi:10.1186/s12904-020-00543-y)
Supplement: Supplementary file 2 — Additional file 2 Backgrounds of participating physicians (n = 22). [file 12904_2020_543_MOESM2_ESM.docx]

| Additional file 2 Backgrounds of participating physicians (n=22) |  |  |
| --- | --- | --- |
|  | n | % |
| Sex |  |  |
| Male | 19 | 86.4 |
| Female | 3 | 13.6 |
| Years of work experience (mean ± standard deviation) | 14.1 ± 3.5 |  |
| Training in a palliative care unit | 12 | 54.5 |
| Participation in the nationwide palliative care education program | 17 | 77.3 |
