# Supplementary material for: Frequency of discussing and documenting advance care planning in primary care: secondary analysis of a multicenter cross-sectional observational study
Source: BMC Palliat Care. 2020 Mar 17;19:32. doi: 10.1186/s12904-020-00543-y (PMC7079526; doi:10.1186/s12904-020-00543-y)
Supplement: Supplementary file 3 — Additional file 3. Prevalence of the ACP discussion and topics discussed in each disease category. [file 12904_2020_543_MOESM3_ESM.docx]

Additional file 3 Prevalence of the ACP discussion and topics discussed in each disease category

|  | Cancer  (n=14) |  | Dementia/frailty (n=58) |  | Neurological disease (n=18) |  | Heart/ vascular disease (n=38) |  | Respiratory disease (n=13) |  | Kidney disease (n=5) |  | Liver disease (n=3) |  |
| --- | --- | --- | --- | --- | --- | --- | --- | --- | --- | --- | --- | --- | --- | --- |
|  | n | % | n | % | n | % | n | % | n | % | n | % | n | % |
| About future ADL declines |  |  |  |  |  |  |  |  |  |  |  |  |  |  |
| Discussed with the patient | 5 | 35.7 | 25 | 43.1 | 3 | 16.7 | 8 | 21.1 | 1 | 7.7 | 1 | 20.0 | 0 | 0.0 |
| Discussed with the family | 0 | 0.0 | 18 | 31.0 | 3 | 16.7 | 2 | 5.3 | 0 | 0.0 | 0 | 0.0 | 0 | 0.0 |
| Documented in medical records | 4 | 28.6 | 20 | 34.5 | 2 | 11.1 | 4 | 10.5 | 1 | 7.7 | 0 | 0.0 | 0 | 0.0 |
| Discussed with the patient and documented in medical records | 4 | 28.6 | 10 | 17.2 | 0 | 0.0 | 2 | 5.3 | 1 | 7.7 | 0 | 0.0 | 0 | 0.0 |
| Discussed with the family and documented in medical records | 0 | 0.0 | 1 | 1.7 | 0 | 0.0 | 0 | 0.0 | 0 | 0.0 | 0 | 0.0 | 0 | 0.0 |
| Discussed with the patient and family and documented in medical records | 0 | 0.0 | 9 | 15.5 | 2 | 11.1 | 2 | 5.3 | 0 | 0.0 | 0 | 0.0 | 0 | 0.0 |
| About future inability to eat |  |  |  |  |  |  |  |  |  |  |  |  |  |  |
| Discussed with the patient | 2 | 14.3 | 15 | 25.9 | 2 | 11.1 | 4 | 10.5 | 1 | 7.7 | 0 | 0.0 | 0 | 0.0 |
| Discussed with the family | 0 | 0.0 | 7 | 12.1 | 2 | 11.1 | 1 | 2.6 | 0 | 0.0 | 0 | 0.0 | 0 | 0.0 |
| Documented in medical records | 2 | 14.3 | 10 | 17.2 | 1 | 5.6 | 2 | 5.3 | 1 | 7.7 | 0 | 0.0 | 0 | 0.0 |
| Discussed with the patient and documented in medical records | 2 | 14.3 | 7 | 12.1 | 0 | 0.0 | 1 | 2.6 | 1 | 7.7 | 0 | 0.0 | 0 | 0.0 |
| Discussed with the family and documented in medical records | 0 | 0.0 | 0 | 0.0 | 0 | 0.0 | 0 | 0.0 | 0 | 0.0 | 0 | 0.0 | 0 | 0.0 |
| Discussed with the patient and family and documented in medical records | 0 | 0.0 | 3 | 5.2 | 1 | 5.6 | 1 | 2.6 | 0 | 0.0 | 0 | 0.0 | 0 | 0.0 |
| About surrogate decision makers |  |  |  |  |  |  |  |  |  |  |  |  |  |  |
| Discussed with the patient | 0 | 0.0 | 13 | 22.4 | 5 | 27.8 | 4 | 10.5 | 1 | 7.7 | 0 | 0.0 | 0 | 0.0 |
| Discussed with the family | 0 | 0.0 | 11 | 19.0 | 5 | 27.8 | 1 | 2.6 | 1 | 7.7 | 0 | 0.0 | 0 | 0.0 |
| Documented in medical records | 0 | 0.0 | 8 | 13.8 | 5 | 27.8 | 4 | 10.5 | 1 | 7.7 | 0 | 0.0 | 0 | 0.0 |
| Discussed with the patient and documented in medical records | 0 | 0.0 | 3 | 5.2 | 0 | 0.0 | 3 | 7.8 | 0 | 0.0 | 0 | 0.0 | 0 | 0.0 |
| Discussed with the family and documented in medical records | 0 | 0.0 | 0 | 0.0 | 0 | 0.0 | 0 | 0.0 | 0 | 0.0 | 0 | 0.0 | 0 | 0.0 |
| Discussed with the patient and family and documented in medical records | 0 | 0.0 | 5 | 8.6 | 5 | 27.8 | 1 | 2.6 | 1 | 7.7 | 0 | 0.0 | 0 | 0.0 |
| About any one topic |  |  |  |  |  |  |  |  |  |  |  |  |  |  |
| Discussed with the patient | 5 | 35.7 | 29 | 50.0 | 7 | 38.9 | 9 | 23.7 | 1 | 7.7 | 1 | 20.0 | 0 | 0.0 |
| Discussed with the family | 0 | 0.0 | 21 | 36.2 | 7 | 38.9 | 2 | 5.3 | 1 | 7.7 | 0 | 0.0 | 0 | 0.0 |
| Documented in medical records | 4 | 28.6 | 23 | 39.7 | 6 | 33.3 | 6 | 15.8 | 1 | 7.7 | 0 | 0.0 | 0 | 0.0 |
| Discussed with the patient and documented in medical records | 4 | 28.6 | 11 | 19.0 | 0 | 0.0 | 4 | 10.5 | 1 | 7.7 | 0 | 0.0 | 0 | 0.0 |
| Discussed with the family and documented in medical records | 0 | 0.0 | 1 | 1.7 | 0 | 0.0 | 0 | 0.0 | 0 | 0.0 | 0 | 0.0 | 0 | 0.0 |
| Discussed with the patient and family and documented in medical records | 0 | 0.0 | 12 | 20.7 | 6 | 33.3 | 2 | 5.3 | 1 | 7.7 | 0 | 0.0 | 0 | 0.0 |
