# Supplementary material for: Frequency of discussing and documenting advance care planning in primary care: secondary analysis of a multicenter cross-sectional observational study
Source: BMC Palliat Care. 2020 Mar 17;19:32. doi: 10.1186/s12904-020-00543-y (PMC7079526; doi:10.1186/s12904-020-00543-y)
Supplement: Supplementary file 4 — Additional file 4. Relationships between physician background factors and the ACP discussion. [file 12904_2020_543_MOESM4_ESM.docx]

|  | Univariate analysis | | | Multivariate analysis | | |
| --- | --- | --- | --- | --- | --- | --- |
|  | OR | 95% CI | p value | OR | 95% CI | p value |
| Male sex | 8.0 | 1.9-33.5 | 0.001 | 6.6 | 1.5-29.3 | 0.012 |
| Clinical practice for > 15 years | 2.1 | 1.3-3.4 | 0.005 | 1.9 | 1.1-3.3 | 0.021 |
| Training in a palliative care unit | 2.1 | 1.3-3.5 | 0.005 | 2.6 | 1.4-4.6 | 0.002 |
| Participation in nationwide palliative care education | 2.1 | 1.0-4.3 | 0.044 | 1.1 | 0.5-2.5 | 0.846 |
| OR: odds ratio |  |  |  |  |  |  |
| 95% CI: 95% confidence interval |  |  |  |  |  |  |

Additional file 4 Relationships between physician background factors and the ACP discussion
